# Sadness might isolate you in a non-smelling world: olfactory perception and depression

**DOI:** 10.3389/fpsyg.2014.00045

**Published:** 2014-02-07

**Authors:** Sylvia Schablitzky, Bettina M. Pause

**Affiliations:** Department of Experimental Psychology, Heinrich-Heine-UniversityDüsseldorf, Germany

**Keywords:** major depression, sadness, odor perception, olfactory sensitivity, bipolar disorder, emotion

## Abstract

Major depressive disorder (MDD) occurs with a high prevalence among mental illnesses. MDD patients experience sadness and hopelessness, with blunted affective reactivity. However, such depressive episodes are also key symptoms in other depressive disorders, like Bipolar Disorder (BPD) or Seasonal Affective Disorder (SAD). Moreover, depressive symptoms can also be found in healthy individuals, but are experienced as less severe or for a shorter duration than in patients. Here, it is aimed to summarize studies investigating odor perception in depression, including depressive states in healthy individuals and patient populations. Odor perception in depression has been assessed with psychophysical methods (olfactory sensitivity, odor identification, and discrimination), and odor ratings (intensity, emotional valence, familiarity). In addition, some studies investigated affective reactions to odors, and physiological and anatomical correlates of odor perception in depression. The summary reveals that MDD is associated with reduced olfactory sensitivity. However, odor identification and discrimination scores seem to be unaffected by depression. The reduced olfactory sensitivity might be associated with a reduced ability to encode olfactory information and a reduced volume of the olfactory bulb. While similar processes seem to occur in healthy individuals experiencing depressive states, they have not been observed in BPD or SAD patients. However, in order to conclude that the reduced olfactory sensitivity is directly linked to depression, it is suggested that studies should implement control measures of cognitive performances or perceptual abilities in other stimulus modalities. It is concluded that the reduced olfactory performance in MDD patients seems to be disorder-, modality-, and test-specific, and that the application of an appropriate olfactory and cognitive test-battery might be highly useful in the differential diagnosis of MDD.

## Introduction

Everybody knows the feeling of sadness as a transient mood state. Sadness is considered to be one of six basic emotions, all of which being experienced in healthy humans independent of culture (anger, disgust, fear, happiness, sadness, and surprise; Ekman and Davidson, 1994). Several brain areas involved in cerebral processing of these emotions are described (Panksepp, 2011; LeDoux, 2012), with sad states being regulated most prominently by the anterior cingulate cortex and the dorsomedial prefrontal cortex (Murphy et al., 2003). But what kind of feelings and behavior differentiate the normal experience of transient sadness from an affective state of depression? The clinical categories and diagnostic criteria for mood and other mental disorders can be assessed via the diagnostic and statistical manual of mental disorders (DSM-5, American Psychiatric Association, APA, 2013).

Whereas in former times (DSM IV; APA, 2000) depression belonged to the category of affective disorders that included unipolar and bipolar depressive disorders, in the present view both disorders are clearly separated from each other (DSM-5, APA, 2013). Among the unipolar depressive disorders, the two most prevalent disorders are Major Depressive Disorder (MDD) and Dysthymia. Both of them share the key symptoms of a depressed mood and a loss of interest or pleasure. However, MDD patients strongly suffer from the depressive mood during at least a 2-weeks period, while in Dysthymia patients, the depressive mood is less severe but lasts at least for 2 years. Bipolar disorders (BPDs) on the other hand, differ from unipolar depressive disorders in the experience of manic (Bipolar Disorder I, BPD I) or hypomanic episodes (Bipolar Disorder II, BPD II), states of abnormally elevated physical or mental activity, usually accompanied by an inflated self-esteem. In BPD patients, the manic phases often alternate with depressive episodes. Both, BPD and MDD, can occur with a seasonal pattern, with depressive episodes regularly reoccurring during fall or winter time.

MDD as well as BPD are described to include one or more major depressive episodes. Therefore, the occurrence of an episode of major depression is not a diagnostic category by itself. The criteria of a major depressive episode are as follows: during 2 weeks nearly the whole time, at least five of the following symptoms have to be present: depressive mood like sadness or hopelessness, reduced interest in activities, significant loss of appetite or increased appetite as well as weight loss or weight-gain without going on a diet, insomnia or hypersomnia, increased psychomotor agitation or retardation, fatigue, feelings of worthlessness, diminished ability to concentrate, and thoughts of suicide. Regarding these symptoms, MDD and BPD are mood disorders affecting functions of motor behavior, perception, memory, cognition, and motivation.

Among all mental disorders, MDD occurs with a high prevalence. The 12-month prevalence in the United States is approximately 7%, with females experiencing 1.5–3-fold higher rates than males. With a 12-month prevalence of 0.6%, BPD has a lower prevalence than MDD (USA, APA, 2013).

There are several theories explaining different aspects of MDD. Cognitive approaches focus on biased information processing and dysfunctional beliefs about the self, the outside world and the future (Beck, 1979). Studies using neuroimaging techniques show alterations of cerebral blood flow and metabolic differences between depressed patients and healthy controls in the amygdala and anatomically related areas of the prefrontal cortex (see Drevets, 2003). Moreover, it is suggested that dysfunctions in the serotonin receptor and other monoaminergic systems could lead to MDD (see Savitz et al., 2009; Savitz and Drevets, 2013). Recently, it has been shown that the deviant serotonergic neurotransmission seems to be responsible for a decoupled cingulated-amygdala-interaction (Pezawas et al., 2005).

Depressive episodes occurring predominately during a particular time of the year (e.g., in the fall or winter) have been termed Seasonal Affective Disorder (SAD). The underlying mechanisms of SAD, MDD, or BPD are supposed to be different, considering the improvement of SAD symptoms, but not MDD or BPD symptoms, through light therapy (exposure to a standard regimen of 10,000 lux cool-white fluorescent light, e.g., Eastman et al., 1998). In SAD, functional deviations within the suprachiasmatic nucleus of the hypothalamus are described (Krout et al., 2002). The suprachiasmatic nucleus is a central structure that mediates behavioral responses induced by the change in the length of daylight.

As mentioned before, alterations of cerebral blood flow and metabolism in the limbic cingulated cortex and prefrontal cortex are consistently observed in MDD patients. Within these brain areas, the amygdala and the ventromedial (orbitofrontal) prefrontal cortex seem to be the most affected in MDD patients (Murray et al., 2011). As well as in emotional processing (LeDoux, 2007), the amygdala is inherently involved in the processing of odor perception (Soudry et al., 2011) and is part of the primary olfactory cortex (Carmichael et al., 1994). From the amygdala olfactory information can be directly transmitted to the orbitofrontal cortex, which is the main neocortical relay for olfactory information (Carmichael et al., 1994; Gottfried, 2006). According to this overlap in odor and emotion processing structures, affective disorders like MDD should accompany alterations of olfactory perception.

Human olfaction has been divided into hierarchical organized levels that are characterized as either primary or secondary. As described by Martzke et al. (1997), olfactory sensitivity is a part of a primary and sensory level of stimulus processing in the olfactory system. However, abilities like olfactory identification, discrimination or odor recognition and odor ratings belong to the secondary and evaluative level of olfaction. The various functions of the olfactory system are to be assessed by suitable methods (Weierstall and Pause, 2012). Most tests for the assessment of olfactory functions fall into three main classes: threshold (absolute sensitivity), identification, and discrimination. Olfactory sensitivity is understood as a measure of the lowest concentration of a particular olfactory stimulus required to activate the receptor neurons resulting in the detection of that odor (Martzke et al., 1997). For the assessment of the odor threshold, a staircase threshold procedure (Bekesy, 1947; Doty, 1991) has been developed that can be used with several odors (Doty and Laing, 2003). This threshold test has been adopted by numerous laboratories (e.g., Pause et al., 2001; Lötsch et al., 2008).

Odor identification is a measure of an individual's ability to perceive and name an odor. Three types are common: first, a simple naming task, prompting the individual to supply a name for a given odor; second, a yes-no odor identification test, in which the participant has to decide whether the odor presented matches a given verbal label or not; or third, a multiple choice odor identification test, with a list of odor names provided for each stimulus. The 40-item, multiple choice University of Pennsylvania Smell Identification Test (UPSIT) is widely used to assess identification performance (Doty et al., 1984b). In the UPSIT, the participant has to choose the odor quality of a given odor out of four verbal descriptors. An odor identification test is also included in the Sniffin' Sticks test battery (Hummel et al., 2007), which is constructed similar to the UPSIT but contains 16 items only.

Odor discrimination is defined as a measure of an individual's ability to differentiate between a set of odors. The most simple form is to state whether two odors are the same or different. However, common tasks involve participants picking out the odd odor out of a series of odors, all of which identical except for one. The only commercially available odor discrimination test is included in the Sniffin' Sticks (Hummel et al., 2007). It comprises 16 items, of which participants are required to choose the odd stimulus out of three given odors.

Psychological attributes of odors are assessed mainly with regard to their intensity, hedonic aspects (pleasantness and unpleasantness) or familiarity. Rating scales can be used to estimate the relative amount of a psychological attribute perceived by an individual. According to Doty and Laing (2003) in chemosensory assessment, two types are popular: category scales, and analog scales. Using category scales, the relative amount of a sensation is signified by indicating which of a series of discrete categories best describes the sensation. Using visual analog scales, the strength of the sensation is indicated by placing a mark along a line that might have descriptors (termed anchors) located at its extremes (e.g., very weak—very strong). Contrary to olfactory sensitivity, odor evaluations are suprathreshold procedures mostly and therefore part of the secondary cognitive evaluative level of the olfactory processing system. For some odors, pleasantness and intensity are closely related psychological dimensions and negatively correlated (Doty, 1975).

In the following review, psychophysical and neurophysiological findings of olfactory performances (sensitivity, identification, discrimination, and odor ratings) in depressed patients (MDD, BPD, SAD) and in healthy individuals experiencing only some depressive symptoms or a transient state of sad mood will be summarized. By expanding this review to healthy people and including the recent literature, this review will add on existing summaries on olfaction and depression (Settle and Amsterdam, 1991; Serby et al., 1992; Atanasova et al., 2008; Burón and Bulbena, 2013).

## Methods

Literature research was based on PubMed (National Center for Biotechnology Information, Bethesda, MDUSA). Only studies investigating distinct groups of MDD, BPD, or SAD patients were included. Mixed samples consisting of BPD and MDD patients in one group or different psychiatric patients in one group were excluded. Further, only publications on chemical perception of standard odors were taken into account. Publications regarding the perception of body odors were not considered, because body odors might be processed by different systems than the olfactory system (Pause, 2012). As it was aimed to focus on the distinct emotional experience of sadness, studies examining olfaction in personality disorders were excluded from the literature search. Finally, effects of odors on mood or therapeutic effect of odors were not considered.

## Findings on olfactory function in depression

Research on olfactory dysfunction in patients with depressive disorders mainly focused on psychophysical assessment of olfactory perception. Particularly, olfactory sensitivity, identification and odor ratings including evaluations of odor characteristics like pleasantness, unpleasantness, intensity or familiarity were investigated. To a lesser extent, patients with depressive disorders have been examined with reference to psychophysiological and neuroanatomical aspects of odor perception, like chemosensory event-related potentials (CSERPs, Pause et al., 2003) or the volume of the olfactory bulb (Negoias et al., 2010). In the following sections, we will review the psychophysics, psychophysiology, and neuroanatomy of olfactory perception in patients with depressive disorders.

### Olfactory sensitivity in depressive disorders

Almost all studies regarding olfactory acuity in MDD indicate that olfactory sensitivity is reduced (corresponding to elevated detection thresholds) in patients, as compared to healthy controls (Table 1). Pause et al. (2001) examined olfactory sensitivity in medicated patients with acute MDD. The Beck's depression score (BDI; Beck et al., 1961) was 28.5 ± 11.4 in the patient group. Olfactory thresholds for eugenol (clove-like odor) and phenyl-ethylalcohol (PEA, rose-like odor) were determined using a two-alternative staircase detection procedure (Bekesy, 1947; Doty, 1991). The study showed reduced olfactory sensitivity in MDD patients compared to healthy controls. Similarly, Thomas et al. (2002) reported slightly (*p* < 0.10) elevated thresholds in a sample of 16 unselected depressives without comorbidity and with a mean BDI-Score of 23.8 ± 9.5. In line with these studies, Lombion-Pouthier et al. (2006) reported sensitivity impairments in patients with severe depression (without any comorbidity) and a BDI-Score of 23.8 ± 5.7. Olfactory perception was assessed by means of the Test Olfactif. The Test Olfactif evaluates olfactory sensitivity using L-carvone and tetrahydrothiopene (forced choice procedure for 5 successive concentrations). Odor detection and identification performance are examined with a panel of 16 odors. Participants have to choose the odor bottle out of four bottles (detection task) and they are asked to choose the correct odor label among a list of four labels (indentification task). Negoias et al. (2010) also showed sensitivity impairments in MDD patients [comorbidities (somatoform disorders, posttraumatic stress disorder and anxiety disorders) were accepted for inclusion] with a mean BDI-Score of 29.7 ± 10.8. All participants were screened for possible cognitive impairments by the mini mental state examination (MMSE, Folstein et al., 1975) and olfactory thresholds were assessed by the Sniffin' Sticks (Hummel et al., 2007) with PEA in 16 dilutions.

**Table 1:** **Summary of studies of olfactory sensitivity in affective disorders**.

**Study**	**Number of participants (f/m) and diagnosis**	**Mean age ± *SD***	**Severity-scores**	**Odorants and test method**	**Olfactory sensitivity**
Gross-Isseroff et al., 1994	C: 9 (8/1)	C: 49,11 ± 4.82	P: 24.11 ± 1.17 (Day 0),	Androstenone, isoamyl acetate, three way forced choice ascending method	Day 0 and Day 21: P = C,
	P: 9 (8/1); MDD	P: 49.00 ± 4.56	11.67 ± 1.13 (Day 21),		Day 42: P > C (isoamyl acetate)
			6.44 ± 0.58 (Day 42, HAM-D)		
Krüger et al., 2006	BPD + ETE: 7 (1/6)	BPD + ETE: 33.9 ± 10.7	BPD + ETE: 0.8 ± 1 (HAM-D) 0.8 ± 1.2 (SRMI)	PEA, Sniffin' Sticks	BPD + ETE > BPD – ETE
	BPD – ETE: 9 (5/4)	BPD – ETE: 46.1 ± 11.6	BPD – ETE: 0.4 ± 0.7 (HAM-D) 1.4 ± 1 (SRMI)		
Lombion-Pouthier et al., 2006	C: 58 (36/22)	C: 38.4 ± 13.96	P: 23.75 ± 5.74 (BDI)	L-carvone, tetrahydrothiopene, Test Olfactif	P < C
	P: 49 (35/14); MDD	P: 43.4 ± 17.54			
Negoias et al., 2010	C: 21 (15/6)	C: 39.62 ± 11.39	P: 29.67 ± 10.84 (BDI)	PEA, Sniffin' Sticks, laterized	P < C
	P: 21 (17/4); MDD	P: 36.86 ± 10.13			
Pause et al., 2001	1. Session: C: 24 (15/9) P: 24 (15/9); MDD	C: 44.2 ± 12.6	C: 4.8 ± 2.5 (BDI)	PEA, eugenol, 2alt.-staircase	P < C
		P: 48.4 ± 13.2	P: 28.5 ± 11.4 (BDI)		
	2. Session: C: 18 (13/5) P: 18 (13/5); MDD in remission	C: 46.6 ± 12.8	C: 4.6 ± 3.4 (BDI)	PEA, eugenol, 2alt.-staircase	P = C
		P: 47.9 ± 13.4	P: 11.5 ± 7.1 (BDI)		
Pause et al., 2005	C: 11 (6/5)	C: 33.0 ± 8.6	C: 2.1 ± 2.3 (BDI)	PEA, menthol, 2alt.-staircase	P < C
	P: 11 (7/4); MDD (drug-free)	P: 32.7 ± 5.5	P: 17.7 ± 6.9 (BDI)		
Postolache et al., 1999	C: 24 (17/7)	C: 42.1 ± 11.8	/	PEA, 2alt.-staircase	P (SAD) = C
	P: 24 (17/7); SAD	P: 42.8 ± 9.7			
Postolache et al., 2002	C: 16 (9/7)	C: 39.0 ± 10.8	/	PEA, 2alt.-staircase	P (SAD) > C
	P: 14(7/7); SAD	P: 42.3 ± 11.5			
Serby et al., 1990, 1992	C: 9 (/)	C: 50 – 59	P: 19.9 ± 1.6 (HAM-D)	Geraniol, forced-choice ascending method	P < C (*p* < 0.1)
	P: 9 (0/9); MDD (drug free)	P: 50 – 59			
Swiecicki et al., 2009	C: 30 (20/10)	C: 35.4 ± 2.1	C: 0.5 ± 0.3 (HAM-D), 1.9 ± 0.5 (BDI)	n-butanol, Sniffin' Sticks	RDD = BP = C
	P: 46 (RDD: 20, BPD: 21)	RDD: 35.7 ± 2.3	RDD: 15.2 ± 1.6 (HAM-D), 27.2 ± 2.8 (BDI);		
		BPD: 14.1 ± 1.0 (HAM-D), 23.2 ± 1.8 (BDI)	BPD: 39.6 ± 2.5		
Thomas et al., 2002	C: 24	/	23.8 ± 9.5 (BDI)	/	*P* < C (*p* < 0.1)
	P: 16 (unselected depressed with major depressive episode)				

f, female; m, male; C, controls; P, patients; MDD, major depressive disorder; HAM-D, Hamilton Depression Rating-Scale; =, no difference between groups; P > C, patients performed better than controls; P < C, patients performed worse than controls; BPD + ETE, bipolar disorder with event triggered episodes; BPD – ETE, bipolar disorder without event triggered episodes; SRMI, Self-Report Manic Inventory; PEA, phenyl-ethylalcohol; BDI, Beck's Depression Inventory; 2alt.-staircase, two-alternative staircase detection procedure; SAD, Seasonal affective disorder; RDD, unipolar recurrent depressive disorder; BPD, bipolar disorder; /, no information available.

Two studies investigated whether the reduced olfactory sensitivity is directly related to the depressive disorder or secondary to the effects of antidepressant drugs. Serby et al. (1990, 1992) examined a sample of 9 MDD patients with a mean Hamilton-Depression-Score (HAM-D; Hamilton, 1960) of 19.9 ± 1.6 under no antidepressant medication. They found a slightly (*p* < 0.10) reduced olfactory sensitivity (elevated olfactory thresholds) to geraniol in patients with MDD compared to healthy controls. Pause et al. (2005) investigated 11 antidepressant drug-free MDD patients. They found significantly elevated olfactory thresholds (PEA and menthol) in patients with moderate MDD (BDI-Score = 17.7 ± 6.9). These results support the conclusion that the decline in olfactory sensitivity in MDD is directly related to the disorder and not mediated by psychiatric treatment.

Gross-Isseroff et al. (1994) investigated olfactory thresholds (androstenone and isoamylacetate) in 9 MDD patients, three times during the course of their psychiatric treatment (HAM-D-Score: Day 0: 24.1 ± 1.2, Day 21: 11.7 ± 1.1 and Day 42: 6.4 ± 0.6). They observed a significant increase in olfactory sensitivity (only isoamylacetate) in MDD patients 6 weeks after initiation of antidepressant drug treatment. This finding is in line with the results from Pause et al. (2001) who showed that after successful medical treatment, sensitivity impairments in MDD patients were reduced. Further, Pause et al. (2001, 2005) and Negoias et al. (2010) reported a significant negative correlation between olfactory sensitivity and the severity of depression.

To our knowledge, only one study did not find any alterations of olfactory thresholds in MDD patients (Swiecicki et al., 2009). In this patient group, the mean HAM-D-Score was 15.2 ± 1.6 and the mean BDI-Score was 27.2 ± 2.8; olfactory thresholds were assessed using the Sniffin' Sticks (n-butanol). Only non-demented (MMSE-score > 24) patients without another psychiatric disorder were included. However, according to the authors, in many of the depressed patients, pharmacotherapy had led to improvement in depressive symptomatology before inclusion to the study. Hence, in line with the findings of Gross-Isseroff et al. (1994) and Pause et al. (2001) successful psychiatric treatment in MDD seems to renormalize olfactory performance.

While the vast majority of studies have investigated odor perception in MDD patients, few studies have examined olfactory sensitivity in related depressive disorders. Swiecicki et al. (2009) reported no differences of olfactory thresholds in patients with BPD, suggesting that sensory aspects of olfactory function cannot serve as a reliable indicator of patients' polarity. Contrasting BPD patients with and without a history of event-triggered episodes, Krüger et al. (2006) showed in a pilot study that olfactory sensitivity (as assessed by the Sniffin' Sticks) was higher in patients vulnerable to emotional stress (with event-triggered episodes, *n* = 7) than in patients without event-triggered episodes (*n* = 9). A healthy control group did not exist in this study.

To our knowledge, there are only two studies on olfactory performance and SAD, however, with conflicting results. In one study, Postolache et al. (1999) found no differences in olfactory thresholds between patients (SAD without comorbidity) and healthy controls or between patients before and after light treatment (exposure to a standard regimen of 10,000 lux cool-white fluorescent light therapy for 45 min twice daily). In the other study, Postolache et al. (2002) observed lower olfactory detection thresholds (a higher olfactory acuity) in SAD patients compared to controls regardless of season.

In summary, the presented evidence shows that olfactory sensitivity is reduced in MDD. The olfactory impairment is directly related to the affective state and is not affected by anti-depressive medication. Furthermore, the decline in sensitivity is related to the severity of MDD. After successful treatment the olfactory dysfunction in MDD patients disappears. Importantly, the decline in olfactory sensitivity seems to be specifically related to MDD, and has not been observed in other depressive disorders like BPD or SAD. The disorder specificity indicates that the reduced olfactory sensitivity is not caused by a general cognitive decline in depressive disorders. However, only one study controlled cognitive performances in MDD patients (Negoias et al., 2010). Therefore, olfactory threshold measurements seem to serve as a differential diagnostic tool and a reduced olfactory sensitivity might play a role as a marker of MDD.

### Olfactory identification and discrimination abilities in depressive disorders

#### Odor identification

Most studies examining olfactory identification abilities in depressive patients have not found differences compared to healthy controls (Table 2). Using the UPSIT, Amsterdam et al. (1987) found no impairments of odor identification ability in a sample of MDD patients with HAM-D-Scores ranging from 18 to 37. Kopala et al. (1994) and Warner et al. (1990) replicated these results, also measuring olfactory identification ability by the UPSIT in MDD patients (Warner et al., 1990), or patients experiencing a major depressive episode (Kopala et al., 1994). They gave no information about the severity of depression. Pause et al. (2003) asked 20 MDD patients (mean BDI-Score = 26.4 ± 9.3) and 20 healthy controls (mean BDI-Score = 3.2 ± 3.2) to identify the odors of PEA and isobutyraldehyde, presented via an olfactometer. It was shown that the identification rates were similar in patients and controls. Assessing patients with severe depression (mean BDI-Score = 23.8 ± 5.7), Lombion-Pouthier et al. (2006) found identification scores, as indicated by the Test Olfactif, to be similar to those in the control group. In line with these results, Swiecicki et al. (2009) reported no alteration of olfactory identification ability, measured by the Sniffin' Sticks in MDD patients. The mean HAM-D-Score was 15.2 ± 1.6 and the mean BDI-Score was 27.2 ± 2.8 in the patient group. Using the Sniffin Sticks as well, Negoias et al. (2010) showed no differences in olfactory identification ability between healthy controls and MDD patients (mean BDI-Score = 29.7 ± 10.8, ranging from 11 to 51). Another study (Naudin et al., 2012) evaluated psychiatric patients during acute episodes of depression and 6 weeks after antidepressant treatment against healthy controls. On the identification task, participants had to identify single odors (*n* = 8) from a list of four descriptors. Regarding the participants' odor identification performances, there was no significant difference among the three groups, considering all odors or each odor independently.

**Table 2:** **Summary of studies of olfactory identification in affective disorders**.

**Study**	**Number of participants (f/m) and diagnosis**	**Mean age ± *SD***	**Severity-scores**	**Odors and test method**	**Olfactory identification**
Amsterdam et al., 1987	C: 51 (34/17)	C: /	P: 18 – 37 (HAM-D)	UPSIT	P = C
	P: 51 (34/17); MDD/BPD II	P: m: 49 ± 14			
		f: 43 ± 13			
Clepce et al., 2010	C: 37 (21/16)	C: /	/ (BDI and SHAPS)	Sniffin' sticks	P < C (acute MDD)
	P: 37 (21/16); MDD	P: m: 48.31 ± 11.95			P = C (in remission)
		f: 47.52 ± 11.33			
Cumming et al., 2011	C: 22 (11/11)	C: 35.5 ± 9.8	P: 16.3 ± 9.5 (BPRS)	UPSIT	BPD < C
	P: 20 (10/10); BPD	P: 34.6 ± 11.3	9.9 ± 8.0 (YMRS)		
			12.0 ± 8.2 (HAM-D)		
Kopala et al., 1994	C: 77 (47/30)	C: 32.5 ± 11.1	/	UPSIT	P = C
	P: 21 (13/8); MDD	P: 37.0 ± 9.6			
Krüger et al., 2006	BPD + ETE: 7 (1/6)	BPD + ETE: 33.9 ± 10.7	BPD + ETE: 0.8 ± 1 (HAM-D) 0.8 ± 1.2 (SRMI)	Sniffin' sticks	BPD + ETE = BPD – ETE
	BPD – ETE: 9 (5/4)	BPD – ETE: 46.1 ± 11.6	BPD – ETE: 0.4 ± 0.7 (HAM-D) 1.4 ± 1 (SRMI)		
Lombion-Pouthier et al., 2006	C: 58 (36/22)	C: 38.4 ± 13.96	P: 23.75 ± 5.74 (BDI)	Test olfactif	P = C
	P: 49 (35/14); MDD	P: 43.4 ± 17.54			
McCaffrey et al., 2000	AD: 20 (13/7)	AD: 74.15 ± 7.86	AD: 20.85 ± 5.22 (MMSE)	Pocket smell test	MDD > AD
	MDD: 20 (11/9)	MDD: 67.55 ± 7.29	MDD: 28.6 ± 1.54 (MMSE)		
Naudin et al., 2012	C: 54 (36/18)	C: 49.5 ± 12.5	C: 2.33 ± 2.3 (MADRS) P (acute MDD): 35.1 ± 4.5 (MADRS)	Identification of odors (*n* = 8) from a list of four descriptors	P(acute MDD) = P(clinically improved) = C
	P: 18 (12/16); MDD	P: 50.1 ± 13.3	P (clinically improved): 9.1 ± 5.6 (MADRS)		
Negoias et al., 2010	C: 21 (15/6)	C: 39.62 ± 11.39	P: 29.67 ± 10.84 (BDI)	Sniffin' sticks	P = C
	P: 21 (17/4); MDD	P: 36.86 ± 10.13			
Oren et al., 1995	C: 21 (16/5)	C: 38 ± 9	P: 29 ± 6 (HAM-D,SAD-Version)	UPSIT	P (SAD) = C
	P: 21 (16/5); SAD	P: 38 ± 9			
Pause et al., 2003	C: 22 (14/8)	C: 48.4 ± 11.9	C: 3.6 ± 3.2 (BDI); 2.6 ± 3.5 (HAM-D)	PEA, isobutyraldehyde, identification of odors from a set of three odors	P = C
	P: 22 (14/8); MDD	P: 47.2 ± 10	P: 25.7 ± 9.4 (BDI), 21.8 ±8.9 (HAM-D)		
Pentzek et al., 2007	C: 30 (24/6)	C: 77.07 ± 6.81	C: 4.5 ± 3.34 (HAM-D), 8.83 ± 3.02 (ADAS-cog);	Sniffin' sticks	A < C
	P: AD: 20 (15/5)	AD: 75.95 ± 9.09	AD: 5.1 ± 4.72 (HAM-D), 25.05 ± 7.57 (ADAS-cog);		A < MDD
	MDD 20 (15/5)	MDD: 73.45 ± 5.61	MDD: 19.05 ± 7.57 (HAM-D), 9.4 ± 3.22 (ADAS-cog)		MDD = C
Postolache et al., 1999	C: 24 (17/7)	C: 42.1 ± 11.8	/	UPSIT	P = C
	P: 24 (17/7); SAD	P: 42.8 ± 9.7			
Serby et al., 1990, 1992	C: 9 (/)	C: 50 – 59	P: 19.9 ± 1.6 (HAM-D)	UPSIT; Yes/No task	UPSIT: P < C
	P: 9 (0/9) MDD	P: 50 – 59			Yes/No task: P = C
Solomon et al., 1998	AD: 20 (12/8)	AD: 74.5 ± 7.77	/	Pocket smell test	MDD > AD
	MDD: 20 (13/7)	MDD: 69.4 ± 7.69			
Swiecicki et al., 2009	C: 30 (20/10)	C: 35.4 ± 2.1	C: 0.5 ± 0.3 (HAM-D), 1.9 ± 0.5 (BDI)	Sniffin' sticks	RDD = BPD = C
	P: 46 (RDD: 20, BPD: 21)	RDD: 35.7 ± 2.3	RDD: 15.2 ± 1.6 (HAM-D), 27.2 ± 2.8 (BDI);		
		BPD: 39.6 ± 2.5	BPD: 14.1 ± 1.0 (HAM-D), 23.2 ± 1.8 (BDI)		
Warner et al., 1990	C: 8	C: 32 (20 – 44)	/	UPSIT	P = C
	P: 6; MDD	P: 37 (28 – 50)			
Zucco and Bollini, 2011	C: 12 (6/6)	C: 39.8 ± 7.1	/	Identification of odors (*n* = 10) from a list of four descriptors	Severe MDD < Mild MDD = C
	P: 12 (6/6) Mild MDD;	P: Mild MDD: 41.3 ± 6.4			
	12 (6/6) Severe MDD	Severe MDD: 41.9 ± 6.2			

f, female; m, male; C, controls; P, patients; MDD, major depressive disorder; BPD II, bipolar disorder II; HAM-D, Hamilton Depression Rating-Scale; UPSIT, University of Pennsylvania Smell Identification Test; =, no difference between groups; P < C: patients performed worse than controls; P > C patients performed better than controls; BDI, Beck's Depression Inventory; SHAPS, Snaith-Hamilton-pleasure-scale; BPRS, Brief Psychiatric Rating Scale; YMRS, Young Mania Rating Scale; BPD + ETE, bipolar disorder with event triggered episodes; BPD – ETE, bipolar disorder without event triggered episodes; SRMI, Self-Report Manic Inventory; AD, Alzheimer's dementia; MMSE, Mini-Mental State Exam; PEA, Phenyl-ethylalcohol; MADRS, Montgomery-Asberg Depression Rating Scale; SAD, seasonal Affective Disorder;ADAS-cog, Alzheimer's disease Assessment Scale; RDD, unipolar recurrent depressive disorder; BPD, bipolar disorder; /, no information available.

In three studies MDD patients' identification performance was compared to the identification performance in patients with Alzheimer's dementia (AD). Solomon et al. (1998) and McCaffrey et al. (2000) applied the Pocket Smell Test, a three-item short version of the UPSIT to 20 MDD and 20 AD patients. In the latter study the patients' cognitive status was assessed by the MMSE, revealing cognitive impairments in AD patients but not in MDD patients. The authors found that depressive patients scored significantly better on the identification test than AD patients, thereby, resembling the performance of healthy controls. Pentzek et al. (2007) investigated odor identification performance by means of the Sniffin' Sticks in 20 AD patients, 20 MDD patients and 30 healthy controls. Whereas MDD patients did not differ from healthy participants in their cognitive status (evaluated by the German version of the Alzheimer's disease assessment scale; Ihl and Weyer, 1993), AD patients showed a significant cognitive decline compared to the two other groups. With respect to the odor identification test, AD patients performed significantly worse than MDD patients and the control group, whereas MDD patients and healthy controls did not differ in their odor identification ability.

Few studies have shown reduced olfactory identification ability in depressed patients. First, Serby et al. (1990, 1992) reported odor identification deficits in patients with MDD (mean HAM-D-Score of 19.9 ± 1.6) using the UPSIT. However, the same authors employed the yes-no identification task and did not show differences between MDD patients and controls. In the yes-no identification task, odors were presented in similar quality pairs (e.g., lemon and orange) and participants were asked to decide, whether the presented odor matched a given verbal label. As compared to the UPSIT, the yes-no identification task might be easier to perform, requiring less cognitive resources. The authors hypothesized that the differences between UPSIT and yes-no performance in depressive patients may be a function of task-specific difficulty, suggesting that the reduced identification performance in MDD might be due to general deficits in cognitive demanding tasks. Assessing odor identification performance by the Sniffin' Sticks in a sample of MDD patients during a depressive episode and in a remitted state, Clepce et al. (2010) showed a significant reduced odor identification score only during the depressive state. In line with Serby et al. (1992), Clepce et al. (2010) attributed the poor identification performance in MDD patients to strong general cognitive impairments. Zucco and Bollini (2011) investigated olfactory identification and olfactory recognition performance in patients with mild MDD and in patients with severe MDD as well as in healthy controls. On the identification task, participants had to smell an odor randomly selected from a set of 10 (aniseed, cinnamon, coffee, garlic, ink, lavender, marsala liquor, mint, petrol, and shoe-polish cream) and had to identify the correct label for each odor (four-alternative-forced choice). The study revealed significantly worse identification performance in the severe MDD group compared to both the mild MDD group and the healthy control group. In addition, olfactory identification performance was significantly correlated with olfactory recognition performance. The authors concluded that the results indicate the suitability of olfactory identification tasks for the assessment of cognitive decline in MDD.

Few studies have examined olfactory identification performance in other depressive disorders than MDD. Oren et al. (1995) examined the odor identification performance (UPSIT) in 21 medication-free patients with SAD, and found that the patients scored as high as the 21 healthy controls. Postolache et al. (1999) applied the UPSIT to 24 SAD patients and 24 matched controls. Even though the UPSIT score did not significantly differ between patients and controls, a negative correlation between the UPSIT score and the score for typical depressive syndromes emerged in depressed patients. This correlation was only observed for right nostril stimulation, but not for left nostril stimulation.

Krüger et al. (2006) examined BPD patients with (*n* = 7) and without (*n* = 9) event triggered episodes. Odor identification performance was assessed by the Sniffin' Sticks and no differences between groups were observed. Swiecicki et al. (2009) investigated olfactory identification performance in 21 BPD patients, using the Sniffin' Sticks, and found no olfactory alterations in the patient group, as compared to healthy controls. A more recent study with 20 BPD patients (without comorbidity; Cumming et al., 2011) observed lower odor identification scores (UPSIT) in BPD patients than in healthy controls. However, the olfactory deficit in the BPD group was significantly less pronounced than in a group of Schizophrenia patients. In this study, participants with IQs < 75 were excluded (evaluated by the Wechsler abbreviated scale of intelligence; Wechsler, 1999).

Summarizing odor identification performances in MDD patients, most studies indicate that patients do not differ from healthy controls. In line with this conclusion, MDD patients have been found to perform significantly better on olfactory identification tasks than AD patients. Few studies reporting reduced olfactory identification scores in MDD point to the possibility that a general cognitive decline in severe depression affects higher order odor processing, such as odor identification. As in MDD, odor identification deficits seem neither to be pronounced in SAD nor in BPD.

#### Odor discrimination

To our knowledge, so far, only one study has investigated odor discrimination ability in MDD patients (Table 3). Using the Sniffin' Sticks Negoias et al. (2010) reported no differences in olfactory discrimination performance between MDD patients and healthy controls. The mean BDI-Score was 29.7 ± 10.8. Odor discrimination performance in BPD was investigated by Krüger et al. (2006). They reported that 7 BPD patients with an event-triggered episode did not differ from 9 BPD patients without event-triggered episodes in their ability to discriminate odors.

**Table 3:** **Summary of studies of olfactory discrimination in affective disorders**.

**Study**	**Number of participants (f/m) and diagnosis**	**Mean age ± *SD***	**Severity-scores**	**Odors and test method**	**Olfactory discrimination**
Krüger et al., 2006	BPD + ETE: 7 (1/6)	BPD + ETE: 33.9 ± 10.7	BPD + ETE: 0.8 ± 1 (HAM-D) 0.8 + 1.2 (SRMI)	Sniffin' Sticks	BPD + ETE = BPD – ETE
	BPD – ETE: 9 (5/4)	BPD – ETE: 46.1 ± 11.6	BPD – ETE: 0.4 ± 0.7 (HAM-D) 1.4 ± 1 (SRMI)		
Negoias et al., 2010	C: 21 (15/6)	C: 39.62 ± 11.39	P: 29.67 ± 10.84 (BDI)	Sniffin' Sticks	P = C
	P: 21 (17/4); MDD	P: 36.86 ± 10.13			

f, female; m, male; C, controls; P, patients; BPD + ETE, bipolar disorder with event triggered episodes; BPD – ETE, bipolar disorder without event triggered episodes; HAM-D, Hamilton Depression Rating-Scale; SRMI: Self-Report Manic Inventory; =, no difference between groups; MDD, major depressive disorder; BDI, Beck's Depression Inventory.

### Odor ratings in depressive disorders

In the following, studies assessing the intensity, emotionality (hedonic profile), and familiarity of odors in depressive disorders will be reviewed.

#### Intensity ratings

Most studies observed intensity ratings of odors not to be altered with MDD (Table 4). In a study by Pause et al. (2001) medicated MDD patients (mean BDI-Score: 28.5 ± 11.4) gave intensity ratings of ten odors using a 7-point scale ranging from 0 to 6. Compared to 24 healthy controls (mean BDI-Score: 4.8 ± 2.5), there were no differences of odor intensity ratings. After successful treatment, 18 MDD patients (mean BDI-Score: 11.5 ± 7.1) and 18 healthy controls were tested again and also showed no differences in intensity ratings. In an unselected sample of 16 patients with an MDD-episode (no diagnosis of the disorder, the episode was related to), Thomas et al. (2002) did not show any differences of odor intensity evaluations compared to a control sample of 24 participants. To obtain ratings of six odors an analog scale was used, grading from 1 (minimal intensity) to 5 (maximal intensity). Testing 5 patients with a MDD-episode in stable remission and 5 controls a second time, Thomas et al. (2002) also reported no group differences. In a study by Pause et al. (2003) 20 MDD patients (mean BDI-Score = 26.4 ± 9.3) and 20 healthy controls (mean BDI-Score = 3.2 ± 3.2) were asked to judge the intensity of PEA and isobutyraldehyde (7-point scale ranging from 0 to 6). No differences between patients and controls were observed. Pause et al. (2005) examined odor intensity ratings in 11 psychotropic non-medicated MDD patients (mean BDI-Score: 17.7 ± 6.9) and 11 control participants. They used a 20 cm analog scale, ranging from not intense at all to extremely intense, to observe the ratings of two odors (PEA and menthol). Groups did not differ with respect to their odor intensity ratings. In a sample of 49 MDD patients (mean BDI-Score: 23.8 ± 5.7), Lombion-Pouthier et al. (2006) also observed similar intensity ratings compared to 58 healthy individuals. Intensity analog scales grading from 0 (low intensity) to 10 (high intensity) were used in order to investigate ratings of 16 odors. Clepce et al. (2010) also reported no differences of odor intensity estimations in 37 MDD patients and 37 healthy controls either in an acute MDD or a remission state of MDD. By means of a 200 mm visual analog scale ranging from 0 (very low intensity) and 200 (very high intensity) participants were asked to rate the intensity of the 16 Sniffin Sticks odors.

**Table 4:** **Summary of studies of odor intensity ratings in affective disorders**.

**Study**	**Number of participants (f/m) and diagnosis**	**Mean age ± *SD***	**Severity-scores**	**Odors and test method**	**Olfactory intensity rating**
Atanasova et al., 2010	C: 30 (12/18)	C: 33.4 ± 9.9	C: 2 ± 2.1 (MADRS)	6 olfactory stimuli, prepared from vanillin (pleasant) and butyric acid (unpleasant) in different concentrations magnitude estimate method	Vanillin (three concentrations)/pleasant: P < C
	P: 30 (12/18); MDD	P: 34.6 ±11.1	P: 36.3 ± 6.3 (MADRS)		Butyric acid (three concentrations)/unpleasant: P > C
Clepce et al., 2010	C: 37 (21/16)	C: /	/: (BDI and SHAPS)	16 odors of the sniffin' sticks, visual analog scale	P = C (in acute MDD and in remission)
	P: 37 (21/16); MDD	P: m: 48.31 ± 11.95			
		f: 47.52 ± 11.33			
Lombion-Pouthier et al., 2006	C: 58 (36/22)	C: 38.4 ± 13.96	P: 23.75 ± 5.74 (BDI)	Test olfactif; rating scale	P = C
	P: 49 (35/14); MDD	P: 43.4 ± 17.54			
Naudin et al., 2012	C: 54 (36/18)	C: 49.5 ± 12.5	C: 2.33 ± 2.3 (MADRS)	Isovaleric acid, PEA; rating scale	2-phenylethanol: acute MDD = clinically improved = C Isovaleric acid: acute MDD > C; clinically improved = C
	P: 18 (12/16); MDD	P: 50.1 ± 13.3	P (acute MDD): 35.1 ± 4.5 (MADRS)		
			P (clinically improved): 9.1 ± 5.6 (MADRS)		
Pause et al., 2001	1. Session: C: 24 (15/9) P: 24 (15/9); MDD	C: 44.2 ± 12.6	C: 4.8 ± 2.5 (BDI)	10 odors; rating scale	P = C
		P: 48.4 ± 13.2	P: 28.5 ± 11.4 (BDI)		
	2. Session: C: 18 (13/5) P: 18 (13/5); MDD in remission	C: 46.6 ± 12.8	C: 4.6 ± 3.4 (BDI)	10 odors; rating scale	P = C
		P: 11.5 ± 7.1 (BDI)	P: 47.9 ± 13.4		
Pause et al., 2003	C: 22 (14/8)	C: 48.4 ± 11.9	C: 3.6 ± 3.2 (BDI), 2.6 ± 3.5 (HAM-D)	PEA, isobutyraldehyde, linear scale	P = C
	P: 22 (14/8); MDD	P: 47.2 ± 10	P: 25.7 ± 9.4 (BDI), 21.8 ±8.9 (HAM-D)		
Pause et al., 2005	C: 11 (6/5)	C: 33.0 ± 8.6	C: 2.1 ± 2.3 (BDI)	PEA, menthol; visual analog scale	P = C
	P: 11 (7/4); MDD (drug-free)	P: 32.7 ± 5.5	P: 17.7 ± 6.9 (BDI)		
Pause et al., 2008	MDD: 9 (0/9)	MDD: 55.1 ± 4.5	SZ: 31.9 ± 7.1 (BPRS)	PEA, isobutyraldehyde, linear scale	SZ = MDD
	SZ: 9 (0/9)	SZ: 33.4 ± 7.9	MDD: 22.9 ± 9 (BDI);		
			40.3 ± 16.4 (HAM-D)		
Thomas et al., 2002	C: 24	/	23.8 ± 9.5 (BDI)	8 odors, rating scale	P = C
	P: 16 (unselected depressed with major depressive episode)				

f, female; m, male; C, controls; P, patients; MDD, major depressive disorder; MADRS, Montgomery-Asberg Depression Rating Scale; SHAPS, Snaith-Hamilton-pleasure-scale; PEA, phenyl-ethylalcohol; P < C, patients rated odors less intense; P > C patients rated odors more intense; =, no difference between groups; BDI, Beck's Depression Inventory; SZ, Schizophrenia patients; BPRS, Brief Psychiatric Rating Scale; /, no information available.

In studies by Atanasova et al. (2010) and Naudin et al. (2012) participants' task was to evaluate the intensity of a pleasant odor and an unpleasant odor that were presented in three different concentrations. Vanillin and butyric acid (Atanasova et al., 2010) as well as PEA and isovaleric acid (Naudin et al., 2012) were used to evaluate the odor intensity, either by means of a magnitude estimate method (Atanasova et al., 2010) or by a 10 cm analog scale, labeled at each end (very low intensity/very high intensity; Naudin et al., 2012). Both studies revealed that MDD patients perceived the unpleasant odor as significantly more intense than the control group. Furthermore, Atanasova et al. (2010) reported the perception of the pleasant odor by the MDD patients as less intense compared to the healthy individuals. After 6 weeks of treatment, Naudin et al. (2012) observed similar odor intensity ratings between MDD patients and healthy controls. It was suggested that presented results might indicate an olfactory anhedonia for pleasant odors and an olfactory alliesthesia for unpleasant odors.

In one study, odor intensity ratings of MDD patients (mean BDI score = 22.9 ± 9.0) were compared to odor intensity ratings of schizophrenia patients (range of intensity ratings: 0 to 6; Pause et al., 2008). Intensity ratings did not differ between patient groups.

In summary, the results of most studies regarding odor intensity ratings in MDD showed evaluations of odor intensity to be unaffected by MDD. However, intensity ratings for highly pleasant or unpleasant odors might be changed in patients experiencing major depressive episodes.

#### Hedonic ratings

In a study by Pause et al. (2001) valence ratings of ten odors were investigated in MDD patients in an acute state of MDD (mean BDI score: 28.5 ± 11.4) and in a remission state after successful medical treatment (mean BDI score: 11.5 ± 7.1; see Table 5 for a summary of results regarding hedonic ratings). To assess ratings of odor pleasantness a 7-point scale ranging from −3 to +3 was used. Valence ratings of nine out of the ten odors were similar in acute state MDD patients and healthy controls. However, citral was perceived as significantly more pleasant by depressive patients. After medical treatment, MDD patients and healthy controls gave similar ratings to all odors. As citral has been discussed to have relaxing and anti-depressant properties, the authors suggested that citral might be perceived as more distinct by depressed patients than by healthy controls. The finding that MDD patients rated the hedonic tone of odors similarly to healthy controls was confirmed in three other studies: Assessing perceived odor pleasantness of seven odors in a sample of unselected MDD patients in an acute state and in a stable remission state, Thomas et al. (2002) also reported no differences between the patients' group and the control group. In order to assess hedonic evaluations analog scales were used ranging from −5 (maximal unpleasant) to +5 (maximal pleasant). Pause et al. (2003) asked 20 MDD patients (mean BDI-Score = 26.4 ± 9.3) and 20 healthy controls (mean BDI-Score = 3.2 ± 3.2) to judge the emotional valence of PEA and isobutyraldehyde (7-point scale: −3 to +3). It was shown that the hedonic judgments were similar in patients and controls. Swiecicki et al. (2009) reported that MDD patients (mean BDI score: 27.2 ± 2.8, mean HAM-D score: 15.2 ± 1.5) evaluated the pleasantness of 16 odors similar to healthy participants. The participants' task was to rate each odor of the Sniffin' Sticks identification test as pleasant, unpleasant or neutral.

**Table 5:** **Summary of studies of hedonic odor ratings in affective disorders**.

**Study**	**Number of participants (f/m) and diagnosis**	**Mean age ± *SD***	**Severity-scores**	**Odors and test method**	**Hedonic odor rating**
Atanasova et al., 2010	C: 30 (12/18)	C: 33.4 ± 9.9	C: 2 ± 2.1 (MADRS)	15 olfactory stimuli, prepared from vanillin (pleasant) and butyric acid (unpleasant) in different concentrations and their mixtures; linear scale	Patients rated unpleasant odors as significantly more unpleasant than controls; 9 out of 15 stimuli were rated as less pleasant by patients
	P: 30 (12/18); MDD	P: 34.6 ±11.1	P: 36.3 ± 6.3 (MADRS)		
Clepce et al., 2010	C: 37 (21/16)	C: /	/: (BDI and SHAPS)	16 odors of the sniffin' sticks, visual analog scale	P = C (in acute MDD and in remission); significant interrelation between anhedonia and hedonic estimates during an acute MDD episode
	P: 37 (21/16); MDD	P: m: 48.31 ± 11.95			
		f: 47.52 ± 11.33			
Cumming et al., 2011	C: 22 (11/11)	C: 35.5 ± 9.8	C: /	Odors of the UPSIT; likert like scale	BPD patients rated odors as more pleasant than controls
	P: 20 (10/10) BPD	P: 34.6 ± 11.3	P: 16.3 ± 9.5 (BPRS)		
			9.9 ± 8.0 (YMRS)		
			12.0 ± 8.2 (HAM-D)		
Lombion-Pouthier et al., 2006	C: 58 (36/22)	C: 38.4 ± 13.96	P: 23.75 ± 5.74 (BDI)	Test olfactif; rating scale	Patients over-evaluate the pleasantness of pleasant odors
	P: 49 (35/14); MDD	P: 43.4 ± 17.54			
Naudin et al., 2012	C: 54 (36/18)	C: 49.5 ± 12.5	C: 2.33 ± 2.3 (MADRS)	8 odors, linear scale	Acute MDD: P rated 5 odors as less pleasant, Clinically improved: two odors were rated to be less pleasant by the patients
	P: 18 (12/16); MDD	P: 50.1 ± 13.3	P (acute MDD): 35.1 ± 4.5 (MADRS)		
			P (clinically improved): 9.1 ± 5.6 (MADRS)		
Pause et al., 2001	1. Session: C: 24 (15/9) P: 24 (15/9); MDD	C: 44.2 ± 12.6	C: 4.8 ± 2.5 (BDI)	10 odors; rating scale	9 odors:P = C; citral was rated to be more pleasant by the patients
		P: 48.4 ± 13.2	P: 28.5 ± 11.4 (BDI)		
	2. Session: C: 18 (13/5) P: 18 (13/5); MDD in remission	C: 46.6 ± 12.8	C: 4.6 ± 3.4 (BDI)	10 odors; rating scale	P = C
		P: 47.9 ± 13.4	P: 11.5 ± 7.1 (BDI)		
Pause et al., 2003	C: 22 (14/8)	C: 48.4 ± 11.9	C: 3.6 ± 3.2 (BDI); 2.6 ± 3.5 (HAM-D)	PEA, isobutyraldehyde, linear scale	P = C
	P: 22 (14/8); MDD	P: 47.2 ± 10	P: 25.7 ± 9.4 (BDI), 21.8 ± 8.9 (HAM-D)		
Pause et al., 2005	C: 11 (6/5)	C: 33.0 ± 8.6	C: 2.1 ± 2.3 (BDI)	PEA, menthol; visual analog scale	Patients tended (*p* < 0.1) to rate odors as less unpleasant than controls
	P: 11 (7/4); MDD (drug-free)	P: 32.7 ± 5.5	P: 17.7 ± 6.9 (BDI)		
Pause et al., 2008	MDD: 9 (0/9)	MDD: 55.1 ± 4.5	SZ: 31.9 ± 7.1 (BPRS)	PEA, isobutyraldehyde, linear scale	SZ = MDD
	SZ: 9 (0/9)	SZ: 33.4 ± 7.9	MDD: 22.9 ± 9 (BDI); 40.3 ± 16.4 (HAM-D)		
Swiecicki et al., 2009	C: 30 (20/10)	C: 35.4 ± 2.1	C: 0.5 ± 0.3 (HAM-D), 1.9 ± 0.5 (BDI);	Odors of the sniffin' sticks	RDD and BPD = C;
	P: 46 (RDD: 20, BPD: 21)	RDD: 35.7 ± 2.3, BPD: 39.6 ± 2.5	RDD: 15.2 ± 1.6 (HAM-D), 27.2 ± 2.8 (BDI);		RDD patients rated less olfactory stimuli as pleasant as compared to BPD patients
			BPD: 14.1 ± 1.0 (HAM-D), 23.2 ± 1.8 (BDI)		
Thomas et al., 2002	C: 24	/	23.8 ± 9.5 (BDI)	8 odors, rating scale	P = C
	P: 16 (unselected depressed with major depressive episode)				

f, female; m, male; C, controls; P, patients; MDD, major depressive disorder; MADRS: Montgomery asberg depression rating scale; BDI: Beck's depression inventory; SHAPS, Snaith-Hamilton-pleasure-scale; BPD: bipolar disorder; BPRS, Brief Psychiatric Rating Scale; YMRS, Young Mania Rating Scale; HAM-D: Hamilton depression scale; =, no difference between groups; UPSIT, University of Pennsylvania Smell Identification Test; PEA, phenyl-ethylalcohol; SZ, Schizophrenia; RDD, recurrent depressive disorder; /, no information available.

Similiar to the finding that certain odors (like citral; Pause et al., 2001) may be perceived as more pleasant in MDD patients, Lombion-Pouthier et al. (2006) observed that MDD patients (mean BDI score: 23.75 ± 5.7) evaluated pleasant odors as more pleasant than healthy individuals. Participants gave ratings of 13 pleasant odors using analog scales graduated from 0 (displeasure) to 10 (pleasure). Another study by Pause et al. (2005) found that drug free MDD patients (mean BDI score: 17.7 ± 6.9) were inclined (*p* < 0.10) to rate odors (PEA and menthol) as less unpleasant than healthy controls (mean BDI score: 2.1 ± 2.3).

However, other studies found depressive patients to rather evaluate odors as more unpleasantness than more pleasant, suggesting that the occurrence of anhedonia in MDD might affect hedonic ratings. Clepce et al. (2010) assessed BDI-Scores as well as Snaith-Hamilton-Pleasure-Scale (SHAPS; Franz et al., 2005) scores. MDD patients (acute and remitted state) and healthy controls rated the pleasantness of the 16 odors of the Sniffin' Sticks using 200 mm visual analog scales ranging from −100 (unpleasantness) to +100 (pleasantness). The study revealed a significant correlation between anhedonia and hedonic estimates during the acute episode of MDD, demonstrating that high depression scores are related to low hedonic estimates of odors.

Atanasova et al. (2010) examined hedonic odor ratings in MDD patients with a mean score of 36.3 ± 6.3 on the Montgomery-Asberg Depression rating scale (MADRS; Montgomery and Asberg, 1979). On a 10 cm analog scale (highly unpleasant/highly pleasant) participants were asked to rate the perceived pleasantness of vanillin (representing a pleasant odor), and butyric acid (representing an unpleasant odor), and binary mixtures of both odors, all in three different concentrations. The study revealed that depressed patients perceived unpleasant odors as significantly more unpleasant than controls. Atanasova et al. interpreted this result as an indicator for olfactory negative alliesthesia in MDD.

Naudin et al. (2012) aimed at determining whether olfactory impairments are state or trait markers of a major depressive episode. They evaluated depressed patients during acute episodes of depression (mean MADRS = 35.1 ± 4.5) and 6 weeks after antidepressant treatment (mean MADRS = 9.1 ± 5.6) against healthy controls (mean MADRS = 2.3 ± 2.3). Hedonic ratings of eight odors (four unpleasant odors, four pleasant odors) were assessed using a 10 cm analog scale (highly unpleasant/highly pleasant). During their acute phase, MDD patients rated five out of the eight odors as less pleasant as controls. The deviant pleasantness ratings were rather observed for pleasant and unpleasant odors than for neutral odors. However, after initiation of psychiatric treatment, only two out of eight odors were still judged as less pleasant by depressive patients.

There are only a few studies available that contrast odor hedonics in MDD patients to another psychiatric population. Pause et al. (2008) found no differences in odor valence ratings between MDD patients (mean BDI score: 22.9 ± 9.0; mean HAM-D score: 40.3 ± 16.4) and schizophrenia patients. In this study, valence ratings (7-point scale: −3 to +3) were obtained for PEA and isobutyraldehyde. Swiecicki et al. (2009) reported that MDD patients (mean BDI score: 27.2 ± 2.8, mean HAM-D score: 15.2 ± 1.5) rated fewer olfactory stimuli as pleasant compared to a BPD group (mean BDI score: 23.2 ± 1.8, mean HAM-D score: 14.1 ± 1.0). Participants were to rate each of the 16 odors of the Sniffin' Sticks identification test as pleasant, unpleasant or neutral. Similar to the outcome that BPD patients seem to judge odors as more pleasant than a comparable patient group, Cumming et al. (2011) found that patients with BPD rated odors (40 odors corresponding to the UPSIT items) as significantly more pleasant than healthy controls. The ratings were judged on a five-point scale (−2 to +2).

In conclusion, odor hedonics are not consistently changed in depressive patients (Table 5). Two studies point to the possibility that MDD patients judge certain odors to be more pleasant than healthy controls (Pause et al., 2001; Lombion-Pouthier et al., 2006). However, the majority of studies found MDD patients to judge the emotional valence of odors either in a normal range or as less positive than healthy controls. This effect has been observed for standard test odors and especially for emotionally negative odors (Atanasova et al., 2010). The hypothesis that depressive patients perceive the hedonic profile of emotionally negative odors as more intense than healthy individuals is supported by the finding that MDD patients report a higher physiological arousal in response to emotionally negative odors than healthy controls (Pause et al., 2000). The findings in MDD patients cannot be generalized to BPD patients, who seem to perceive odors as more pleasant than healthy controls.

#### Familiarity ratings

To our knowledge, there are only four studies concerning ratings of odor familiarity in MDD patients (Table 6). There are no studies with respect to familiarity ratings in BPD or SAD. Thomas et al. (2002) found slight differences between a sample of 16 unselected patients suffering from an acute MDD-episode without comorbidity and 24 healthy controls. Familiarity ratings of odors (dried fish, parmesan cheese, gyran, alpha-methylnaphtylketone, coffee, and vanilla) were assessed by means of visual analog scales ranging from −5 (most unfamiliar) to +5 (most familiar). Depressed patients tended to rate vanilla (*p* = 0.051) and dried fish (*p* = 0.099) to be less common than healthy controls. Five MDD patients in a stable remission state of MDD were retested. Compared to 5 retested controls, patients rated odor familiarity in a similar way. Pause et al. (2005) examined 11 antidepressant drug-free MDD patients (mean BDI-Score: 17.7 ± 6.9) and 11 controls (mean-BDI-Score: 2.1 ± 2.3). To assess odor familiarity ratings, they used visual analog scales (anchor: familiar—unfamiliar) and presented PEA and menthol as odors. Groups did not differ in familiarity ratings of either PEA or menthol. Atanasova et al. (2010) assessed odor familiarity ratings in 30 MDD patients (mean MADRS-Score: 36.3 ± 6.3) and 30 healthy controls. They used a 10 cm analog scale, labeled at each end (unfamiliar odor and very unfamiliar odor), for ratings of vanillin and butyric acid. They found no differences between groups for either odor in familiarity evaluations. Naudin et al. (2012) examined odor familiarity ratings in 18 MDD patients and a control group (*n* = 54). They used analog scales for the ratings and assessed ratings again after MDD patients in remission. For all odors, except vanillin, the authors reported no group differences in odor familiarity ratings. Vanillin was evaluated as less familiar by MDD patients and patients in remission as compared to healthy controls.

**Table 6:** **Summary of studies of odor familiarity ratings in affective disorders**.

**Study**	**Number of participants (f/m) and diagnosis**	**Mean age ± *SD***	**Severity-scores**	**Odors and test method**	**Odor familiarity rating**
Atanasova et al., 2010	C: 30 (12/18)	C: 33.4 ± 9.9	C: 2 ± 2.1 (MADRS)	15 olfactory stimuli, prepared from vanillin (pleasant) and butyric acid (unpleasant) in different concentrations and their mixtures; linear scale	Vanillin and butyric acid: P = C
	P: 30 (12/18); MDD	P: 34.6 ±11.1	P: 36.3 ± 6.3 (MADRS)		
Naudin et al., 2012	C: 54 (36/18)	C: 49.5 ± 12.5	C: 2.33 ± 2.3 (MADRS)	8 odors	7 odors: P = C; vanillin: P < C
	P: 18 (12/16); MDD	P: 50.1 ± 13.3	P (acute MDD): 35.1 ± 4.5 (MADRS)		
			P (clinically improved): 9.1 ± 5.6 (MADRS)		
Pause et al., 2005	C: 11 (6/5)	C: 33.0 ± 8.6	C: 2.1 ± 2.3 (BDI);	PEA, menthol; visual analog scale	P = C
	P: 11 (7/4); MDD (drug-free)	P: 32.7 ± 5.5	P: 17.7 ± 6.9 (BDI)		
Thomas et al., 2002	C: 24	/	23.8 ± 9.5 (BDI)	8 odors, rating scale	Vanilla: P < C, (*p* = 0.051); dried fish: P < C (*p* = 0.099); patients in stable remission = C
	P: 16 (unselected depressed with major depressive episode) and 5 retested patients in stable remission				

f, female; m, male; C, controls; P, patients; MDD, major depressive disorder; MADRS, Montgomery-Asberg Depression Rating Scale; PEA, phenyl-ethylalcohol; =, no difference between groups; P < C, patients rated odors less intense; BDI, Beck's Depression Inventory; /, no information available.

In sum, ratings of odor familiarity do not seem to be strongly altered in MDD.

### Affective reactions to olfactory stimuli in MDD

Only two studies investigated emotional reactivity or affective states after odor exposure. Steiner et al. (1993) investigated valence ratings and facial expressive features (assessed by observer ratings with regard to the quality, strength and duration of facial expressions) in 21 MDD patients as an indicator for affective reactions to olfactory stimuli (rose oil and amyl acetate representing pleasant odors and butyric acid and methyl mercaptan representing unpleasant stimuli). The patient sample was found to display reduced facial expressions while presenting pleasant odors and to show reduced durations of facial expressions in response to either pleasant or unpleasant odors. That was contrary to the valence ratings that did not differ between patients and healthy controls.

In another study by Pause et al. (2000), affective reactions to olfactory and visual stimuli (emotional scenes) were assessed in 26 MDD patients (mean BDI-Score: 29.4 ± 11.4) and 26 healthy controls (mean BDI-Score: 4.7 ± 2.5). Participants were to describe their emotional reaction to 10 odors (pleasant, unpleasant and neutral) and 20 pictures on three dimensions (valence, arousal, and dominance) by means of the self-assessment-manikin (Bradley and Lang, 1994). The study revealed higher arousal in MDD patients while presenting negative stimuli for all stimulus modalities.

In sum, both studies indicate that emotional reactions to odors are altered in MDD patients, irrespective of odor evaluation strategies. However, elevated affective reactions to emotionally negative odors do not seem to be modality-specific.

### Psychophysiology and neuroanatomy of olfaction in MDD

The data presented in the previous section indicate that olfactory sensitivity is reduced in depressed patients. Therefore, and following the annotations by Martzke et al. (1997), odor perception in depression seems to be altered on an early sensory processing level. Other perceptual performances, like odor identification and discrimination, do not seem to be strongly altered in MDD patients, indicating that the cognitive-evaluative level of olfactory processing is not impaired in depression.

By means of event-related potential (ERP) analysis, Pause et al. (2003) investigated olfactory, visual, and emotional stimulus processing in MDD patients and in healthy controls. Patients were examined at the beginning of their therapy and after successful medical treatment. Pause and colleagues focused on whether olfactory function in depression is disturbed in a modality-specific manner. Within the ERP, early and late potentials were analyzed. While early potentials, such as the P2, are related to early pre-attentive stimulus encoding, late potentials, such as the P3 and the late positive Slow Wave (pSW), are rather related to late evaluative stimulus processing. The study revealed that MDD patients responded to odors with reduced early (P2) and late (P3-1) potential amplitudes. In response to colors, and emotional slides they showed reduced late potential amplitudes only (colors: P3 and pSW; emotional slides: pSW). After successful psychiatric treatment, the event-related potentials to either stimuli did not differ between groups. The authors discuss the reduction of the early potential amplitudes of the chemosensory ERP in MDD patients to reflect a modality-specific reduction in the ability to encode basic olfactory information on an early level of sensory processing. This interpretation is in line with the data demonstrating a reduced olfactory sensitivity in MDD patients. However, the reduction of the late positive potentials in response to colored and emotional slides might have been related to the non-modality specific effect of a reduced late evaluative stimulus processing in MDD patients.

In another ERP study, Pause et al. (2008) contrasted olfactory and visual stimulus (colored slides) processing in 9 MDD patients and 9 Schizophrenia patients (all males). In response to odors (PEA and isobutyraldahyde), MDD patients showed longer latencies of all ERP components than Schizophrenia patients. Additionally, the amplitude of the pSW in response to colors was larger in MDD patients than in Schizophrenia patients. These results indicate that the reduced olfactory processing capacities (as shown in longer latencies or reduced amplitudes) in MDD patients are modality-specific and prominent in comparison to healthy individuals and also in comparison to other psychiatric patient groups.

Negoias et al. (2010) assessed olfactory function and the volume of the olfactory bulb (OB) in patients with acute MDD. Participants underwent measures of odor threshold, discrimination and identification using the Sniffin' Sticks test battery in a lateralized fashion. OB volumes were calculated by manual segmentation of acquired T2-weighted coronal slices according to a standardized protocol. The study revealed that MDD patients had a significantly lower olfactory sensitivity and smaller OB volumes as compared to healthy controls. There were no group differences for olfactory discrimination and identification scores. A significant correlation between OB volumes and odor thresholds was observed for the left nostril: the lower the olfactory sensitivity, the smaller the OB volume. Additionally, Negoias et al. (2010) found a significant negative correlation between olfactory bulb volume and depression scores (BDI).

In sum, the data indicate that olfactory stimulus processing is altered on an early sensory processing level in patients with acute severe MDD. This olfactory dysfunction seems to be modality- and disorder-specific. Furthermore, the reduced capacity to encode olfactory information in MDD seems to be accompanied by higher olfactory thresholds and smaller OB volumes. Whether or not olfaction is impaired on the later cognitive-evaluative level seems to depend on the progress of general cognitive decline during depressive episodes, but seems not to be depend on the stimulus modality.

### Olfactory performance in healthy individuals with depressive symptoms

In the following section, studies will be outlined, which investigated healthy individuals scoring high on some depressive symptoms but not fulfilling the diagnostic criteria for MDD or any other psychiatric disorder.

Satoh et al. (1996) examined odor ratings in Japanese elderly participants (mean age in men: 73.2 ± 6.0; in women: 72.4 ± 5.9). Odor ratings were obtained for the 40 odor items of the UPSIT. As a main result, it was found that elderly men with increased depression scores (assessed by the self-rating depression scale; Zung, 1965) rated odors to be weaker than their non-depressive counterparts.

In another study (Economou, 2003) smell identification performance (UPSIT) was investigated in elderly Greek people, ranging in age from 49 to 88 years. Correlations between the UPSIT and the BDI-II score were not significant, suggesting that in elderly healthy individuals, a small number of depressive symptoms does not affect odor identification.

Olfactory discrimination scores were assessed in young adults (mean age: 19.3 ± 1.6) by Goel and Grasso (2004). The olfactory discrimination test included 7 items and was based on five different blends of commercially available lavender oil. Participants were to indicate, whether a certain blend was the same, stronger, or weaker than another blend, briefly presented before. All participants rated their depressive symptoms on the BDI manual. Whereas overall olfactory performance was not related to the BDI score, participants with higher BDI scores solved two out of the seven items better than participants with a lower BDI score.

Pollatos et al. (2007a) examined 48 participants who reported a small number of depressive symptoms (BDI score < 10). Olfactory sensitivity and discrimination performance were assessed by means of Sniffin' Sticks (odor for the threshold test: n-butanol). Concerning olfactory sensitivity, the degree of depressive symptoms was inversely correlated to the olfactory threshold score: The higher the number of depressive symptoms the lower the olfactory sensitivity. However, olfactory discrimination performance was not related to the degree of depressive symptoms. These results correspond to data of studies concerning olfactory sensitivity in patients with MDD that reported elevated thresholds in MDD patients.

Pouliot et al. (2008) investigated the relation between olfactory perception (odor detection, identification and ratings of odor pleasantness and intensity) in 32 healthy menopausal women. Anhedonia, one symptom frequently occurring in MDD, was assessed by the Physical Anhedonia Scale (Chapman et al., 1976). Women below or equaling the median anhedonia score of 14 were classified as low-anhedonic and women with a higher anhedonia score than 14 were assigned to the high-anhedonia group. Participants underwent the European test of olfactory capabilities (ETOC; Thomas-Danguin et al., 2003). The test consists of an odor detection task and an odor identification task using a panel of 16 odors. Firstly, Participants are asked to detect the odor bottle out of four bottles (three bottles that are not holding any odor) and, secondly, to identify the detected odor by choosing a label out of four given labels. Olfactory performances of detection (an indicator of olfactory sensitivity) and identification are integrated in one olfactory performance score. Additionally, in the study by Pouliot et al. (2008) pleasantness and intensity of the ETOC-odors were rated by the participants on a 9 point scale. Pouliot et al. (2008) reported that high-anhedonic menopausal women had a worse olfactory function than women with a lower anhedonia score. Women in the low anhedonia group rated more odors as pleasant and as neutral than as unpleasant. Moreover, it was observed that the anhedonia score correlated negatively with the perceived odor pleasantness.

Conflicting results were obtained in a study conducted by Scinska et al. (2008). In a sample of non-clinical older adults (mean age: 63.0 ± 1.1), the relation between depressive symptoms and olfactory performance (odor detection thresholds and odor identification ability) was investigated. Depression scores were assessed by the Geriatric Depression Scale (GDS; Yesavage, 1988). Depending on the GDS-Score, participants were classified as depressed (GDS-Score > 5) or as non-depressed (GDS-Score < 5). In order to assess olfactory performance, Sniffin' Sticks were used and the odor for the threshold test was n-butanol. The study revealed that depressive symptoms were not related to any measurement of olfactory performance. However, the authors found that age was significantly correlated with both olfactory measures; as expected older participants performed worse on the olfactory tests.

In sum, three out of six studies (Satoh et al., 1996; Pollatos et al., 2007a; Pouliot et al., 2008) reveal that the experience of depressive symptoms in healthy individuals affects olfactory performances. All of these studies found scores of odor sensitivity (as assessed by intensity ratings, odor detection performances and threshold tests) to be reduced in individuals with depressive symptoms. However, two studies investigated odor perception (identification and sensitivity) in older adults and failed to establish a relation between olfaction and depression scores (Economou, 2003; Scinska et al., 2008). As olfactory functions have been repeatedly reported to decline with age (e.g., Doty et al., 1984a; Hummel et al., 2007), in these two studies, effects of age might have overshadowed effects of depression, resulting in overall low olfactory performances, which are unlikely to be further reduced by psychiatric symptoms. In addition, one study (Goel and Grasso, 2004) indicated that olfactory discrimination performance for certain odors might even be increased during depressive mood states.

### Olfactory performance in healthy individuals with transiently experienced depression-like feelings

To our knowledge, there are only two studies investigating odor perception in healthy participants who transiently experience depression-like feelings.

Laudien et al. (2006) considered symptoms of learned helplessness as a mood state similar to symptoms occurring in depression. The term “helplessness” has been adopted to denote a negative emotion, which is characterized by lack of control, significant negative expectancies for the future and deterioration of cognitive performance (e.g., Hiroto and Seligman, 1975). By means of ERP analysis, olfactory and auditory stimulus processing was investigated in healthy individuals who transiently experienced helplessness or were in a neutral mood state. In order to induce helplessness, participants were exposed to uncontrollable failure in an unsolvable face-classification task. Odors (PEA and menthol) were presented by an olfactometer. While experiencing helplessness, participants' responses to odorous stimuli were attenuated at an early processing stage: the amplitudes of P2 and P3-1 were smaller and the latencies of N1, P2 and P3-1 were longer. Effects were only shown for the olfactory modality and not for the auditory modality. Results indicate that the CSERP displays transient mood effects, resembling the CSERP effects in MDD patients (Pause et al., 2003).

Pollatos et al. (2007b) investigated olfactory sensitivity and olfactory discrimination ability by Sniffin' Sticks (odor for the threshold test: n-butanol) as well as perceived pleasantness and intensity of n-butanol by a 9 point scale in 32 healthy participants. Prior to the olfactory testing, participants underwent a procedure of emotion induction by presenting pleasant, unpleasant and neutral pictures from the IAPS (International Affective Picture System; the center for the study of emotion and attention). Results show that olfactory sensitivity was decreased after presentation of unpleasant pictures, and only in male participants were olfactory thresholds increased after viewing pleasant pictures as well. Olfactory discrimination ability did not show any alterations in dependence of the emotional state condition. The authors concluded that negative emotional experience is accompanied by a reduced olfactory sensitivity. They suggested that, in addition, odor perception in men is strongly interfered with arousing states.

The studies by Laudien et al. (2006) and Pollatos et al. (2007b) show that even transient experiences of negative mood or helplessness affect odor perception at a sensory level. As in depressive patients, threshold values are increased during negative mood states, but tests involving cognitive performance, like odor discrimination, are not affected by a transient mood decline (Pollatos et al., 2007b). Moreover, as in depressive patients, central nervous processing of odors in helpless individuals is attenuated at an early processing stage, related to stimulus encoding, but unchanged at a late processing stage, related to cognitive odor evaluation.

## Discussion

### Methodological considerations on olfactory assessment and their implications for study differences

Obviously, measuring olfaction in patients with depressive disorders needs to be reliable and valid. Thus, olfaction as well as depression should be assessed with unambiguous measurements.

Measuring olfaction requires the standardization of context variables inherently linked to the chemical senses. Temperature and air humidity are factors that can alter the evaporation rate of odors (Mozell et al., 1986) and should thus be controlled in tests on olfactory performance. Most volatile chemicals stimulate the olfactory as well as the trigeminal nerve. Stimulating the trigeminal nerve produces stinging, burning, tickling, warm, or cold sensations. Regarding olfactory acuity, odors that only stimulate the olfactory nerve (nervus olfactorius) should be used in olfactory testing. Odors, for example n-butanol, which do not exclusively stimulate the first cranial nerve (nervus olfactorius), can also produce sensations in anosmic individuals. PEA or vanillin may be more suitable in olfactory acuity assessment because they seem to be nearly pure olfactory stimulants (Doty et al., 1978). Further, it should be considered whether participants should take a natural sniff in the olfactory test procedure. Berglund et al. (1986) and Laing (1983) showed that olfactory threshold assessment using an external constant airflow produced more elevated thresholds than olfactory assessment with natural sniffing. In addition, sniffing seems to be necessary for the neuronal priming of odor processing within the olfactory bulb (Gerkin et al., 2013). Several procedures have been published which have been used in the measurement of olfactory performance (e.g., number of items, ascending or descending stimulus presentation in threshold tests). These test characteristics have been reviewed elsewhere (Doty, 2006, 2007). In general, implementing a standard test procedure, comparable between different studies, seems to be advantageous. Besides olfaction-specific requirements, performance testing relies on general test characteristics. For example, in olfactory testing the item difficulty is rarely considered. However, in olfaction, easy items (tasks, which are easy to solve in the general population) may be useful in testing relatively strong deficits in elderly individuals or neurologic or psychiatric patients, while difficult items (tasks, which are difficult to solve in the general population) seem to be more suitable in measuring performance differences in healthy young individuals (Weierstall and Pause, 2012).

As already mentioned, functions of human olfaction can be characterized as either primary (olfactory sensitivity) or secondary (olfactory identification, discrimination or recognition). The distinction of human olfaction as either primary or secondary indicates that one single test of olfactory performance might not be a sufficient marker of olfactory functioning (Martzke et al., 1997). However, olfactory sensitivity performance can be altered by centrally mediated events known as top-down processing, and furthermore, the interpretation of secondary olfactory functions (e.g., identification or discrimination) is contingent upon available data about the intactness of the primary sensory systems (e.g., intact olfactory acuity). Many studies of olfactory performance do not regard the necessity to obtain measures on primary and secondary olfactory functions in order to judge olfactory abilities in patients.

Besides psychophysical test procedures, electrophysiological measures, like CSERPs, are promising tools aiding to differentiate primary from secondary odor processing. Research on the relation between olfaction and depression, has demonstrated that early sensory odor processing is attenuated in MDD patients and in healthy individuals experiencing helplessness, while late evaluative odor processing is not affected (or only affected in a modality non-specific manner). These findings correspond to studies showing that olfactory sensitivity is reduced in MDD patients, while odor discrimination and odor identification often remains unaffected.

Similar to the considerations on the tests of olfaction, also the tests of depression need to be appropriate. Thus, in patient studies, it is required to assess the distinct type of depressive disorder and the severity of the depressive symptoms. While most studies include measures of symptom severity (e.g., HAM-D, or BDI), some studies lack a precise diagnosis of the depressive symptoms. Major depressive episodes might occur in MDD, in BPD, in SAD or mood disorders, which are due to other medical conditions (e.g., multiple sclerosis, stroke, hypothyroidism). However, the foregone summary reveals that there are disorder specific alterations of olfactory perception. Therefore, in depression research, it is a prerequisite to specify the differential diagnosis.

A general cognitive decline that occurs during a depressive episode has been suggested to be the underlying process of impaired olfactory identification ability showed by some studies that examined MDD patients. However, cognitive impairments should be considered in olfactory testing in general. For example, memory, attention, executive functions, or language deficits can affect olfactory identification or discrimination performance. The amount of influence by these factors is partly task-dependent: whereas olfactory discrimination tests vary with the amount of attentional resources and working memory performance, olfactory identification tasks, vary with language skills, semantic memory, and executive functions (Doty and Laing, 2003; Schubert et al., 2013; Zucco et al., 2014). Furthermore, the familiarity of an odor can lead to increased discrimination performance (Rabin, 1988). However, olfactory sensitivity seems to be rather unaffected by cognitive factors. According to Hedner et al. (2010) cognitive factors like executive functioning, semantic memory, and episodic memory are unrelated to odor threshold scores. Thus, in order to make sure that alterations in olfactory perception are directly related to the disorder (and it's specific neurological underpinnings) and not secondary to cognitive or motivational deficits, appropriate control conditions need to be implemented. Such control conditions should consist of cognitive tests (most importantly assessing short-term memory, and attentional capacities) or control tests which assess perceptional performances in other modalities than olfaction (Pause et al., 2003).

In summary, there are some methodological considerations which should be taken into account in investigating olfactory performance in depressive disorders. The considerations refer to the kind of olfactory test, which might be most suitable and to the precise measurement of the kind of depressive disorder. To control influences of cognitive or motivational factors on olfactory performance (especially odor identification and odor discrimination), cognitive functioning should be evaluated. As summarized above, the alterations in olfactory performances are specific to the depressive disorder (e.g., MDD or BPD), therefore, direct comparisons of sensory performances between and within psychiatric populations are recommended (see e.g., Pentzek et al., 2007; Pause et al., 2008).

### General implications

The literature on olfaction in depressive disorders and healthy people experiencing a negative mood state has been summarized. Regarding olfactory sensitivity in MDD, almost all studies show elevated olfactory thresholds in patients as compared to healthy controls. The olfactory impairments can be observed whether or not patients are treated with antidepressants, indicating that the reduced odor sensitivity is directly related to the depressive disorder. Furthermore, the sensitivity impairments disappear with successful treatment of MDD. Accordingly, two studies reveal that severity of MDD and reduced olfactory sensitivity are significantly correlated. The decline in olfactory sensitivity seems to be specific for MDD and has not been observed in BPD or SAD.

Olfactory identification ability in MDD was found to be unaffected in most of the studies. However, some studies found reduced odor identification performances in patients with severe MDD. It is likely that the worse odor identification performance in these MDD patients is due to a general cognitive deficit, which accompanies severe depressive symptoms. Due to the limited evidence, the degree of olfactory identification performance in BPD and SAD remains unclear. There is only one study (Negoias et al., 2010) investigating discrimination ability in MDD, revealing no differences between patients and control group.

Evaluations of odor characteristics are assessed with regard to intensity, hedonic aspects or familiarity. Odor intensity ratings seem to be unaffected by MDD. However, intensity ratings for highly pleasant or unpleasant odors might be changed in MDD patients. Most studies reveal the emotional valence of odors to be rated in a normal range or as less positive in MDD patients as compared to healthy participants. This is in line with the findings that MDD patients report a higher arousal in response to emotionally negative odors than healthy participants. In contrast to MDD patients, BPD patients seem to judge odors as emotionally more positive than healthy controls. Familiarity ratings seem not to be altered in MDD.

Regarding the psychophysiology of olfaction, early potential amplitudes of the CSERP in MDD patients are reduced in a modality-specific manner. Further, regarding the neuroanatomy of olfaction in MDD, patients showed smaller OB volumes compared to healthy controls and reduced olfactory sensitivity was negatively correlated with the OB volume. Both findings indicate that odor perception in MDD is altered at an early sensory processing level.

Similar to depressive patients, healthy individuals with depressive symptoms as well as individuals experiencing a transient negative mood seem to show a reduced olfactory sensitivity, whereas odor identification or discrimination appears to be unchanged. In addition, early sensory odor processing, as indexed by CSERP analysis is attenuated in MDD patients as well as in healthy individuals experiencing helplessness. These findings indicate that similar neurophysiological processes appear to modulate the effects of negative mood or depression on the olfactory system.

Altogether, reviewed data show that odor perception in MDD and healthy but sad individuals is altered on an early sensory processing level. Alterations of olfaction on a later cognitive-evaluative level seem to vary with the magnitude of a general cognitive impairment during depressive episodes. These findings have practical and theoretical implications: First, as even healthy individuals experiencing sad mood show reduced olfactory performances it is highly probable that alterations in odor perception precede the manifestation of a depressive disorder. Therefore, olfactory tests could be useful to be added to the early diagnosis of depressive symptoms during the development of a depressive disorder. Furthermore, as the reduced olfactory performance in MDD patients seems to be disorder-, modality-, and test-specific, the application of an appropriate olfactory and cognitive test-battery might be highly useful in the differential diagnosis of MDD (as compared to other disorders which also include the manifestation of depressive episodes).

Second, it is likely that structures of the primary olfactory cortex are affected during depressive experiences. Primary cortical processing structures of the olfactory system are the OB and its direct project areas (the anterior olfactory nucleus, the piriform cortex, the anterior cortical nucleus of the amygdala, the periamygdaloid cortex and the anteromediate part of the entorhinal cortex). The amygdala, especially the anterior cortical nucleus, receives direct information from the OB (see Carmichael and Price, 1994; Cleland and Linster, 2003).

Rats with excised OBs (OB rats) are proposed to be an animal model of depression. It is observed that OB rats show behavioral, neurotransmitter, immune and endocrine changes similar to patients with depression. Additionally, behavioral changes in the OB rat can be treated by antidepressants given chronically (Richardson, 1991). Regarding behavioral changes, OB rats show increased locomotor activity in a novel environment, impaired spatial learning and taste aversion learning. In addition, they show increased reactivity to stressors, which is related to higher levels of adrenocorticotrophic hormone (see Kelly et al., 1997; Harkin et al., 2003). The alterations of the physiology and behavior after bulbectomy are not caused by the loss of smell, as peripherally induced anosmia does not generate depression like symptoms (Song and Leonard, 2005). It is suggested that the bulbectomy disrupts the limbic circuit responsible for flexible modulating of behavior. Probably most important, after bulbectomy the tonic inhibition of amygdala activity through the OB is reduced, resulting in a dysinhibition of the amygdala (McNish and Davis, 1997; Harkin et al., 2003). The amygdala is basically involved in the processing of emotional signals of threat and fear (LeDoux, 2007), and further plays a central role in the physiopathology of depressive disorders (Hamilton et al., 2012). Processing negative stimuli is accompanied by hyperactivity of the amygdala (see Soudry et al., 2011). Following these considerations, reduced olfactory sensitivity might be due to dysfunctions of the OB in depressive patients (Lu and Slotnick, 1998) and additionally, an impaired OB can cause an intensified experience of sadness and fear via disinhibition of the amygdala (Pause et al., 2001).

Individuals experiencing depression show a loss of interest or pleasure in nearly all activities and describe their mood to be sad, hopeless or discouraged. Their emotional experience is generally blunted, involving reduced experiences of positive as well as of negative emotions. Here, we conclude that the reduced emotionality during depressive states is accompanied by a reduced olfactory experience. It is hypothesized that similar neuronal networks are responsible for the attenuated olfactory and emotional experience. The olfactory environment is usually low in distinctiveness and in general only few smells reach our awareness. Therefore, it has been postulated that, in everyday life, olfaction is a rather implicit sense (Köster, 2002). Considering, that a reduced olfactory sensitivity contributes to a still lesser experience of environmental smells, leads to the assumption that sadness might not only to isolate individuals in terms of their emotional belongingness, but also might isolate them with regard to reduced sensory (olfactory) experiences.

### Conflict of interest statement

The authors declare that the research was conducted in the absence of any commercial or financial relationships that could be construed as a potential conflict of interest.
